# How to Sleep

**Published:** 1873-04

**Authors:** 


How to Sleep.—We are often asked for a prescription for preternaturally wakeful persons. The "high pressure" principle on which many of our business men work their brains, and abuse their bodies, begets an irritable condition of the nerves, and a morbid state of mind, very antagonistic to quiet and refreshing sleep. Such persons will often go to bed weary and exhausted, but cannot sleep; or sleep dreamily and fitfully; or lie awake for hours, unable to sleep at all. We have tried many expedients to induce sleep with more or less success, and have read many recipes which proved better in theory than in practice. The very best method we have yet discovered is that of counting. Breathe deeply and slowly (without any straining effort) and, with every expiration, count one, two, three, etc., up to a hundred. Some persons will be asleep before they can count fifty in this manner. Others will count ten, twenty, or thirty, and then forget themselves and cease counting. In such cases always commence again at once. Very few persons can count a hundred and find themselves awake; but should this happen, repeat the dose until cured.—*From Science of Health.*

Professor Goltz, of Konigsberg, in his experiments upon the nervous centre of frogs, finds that if you take out the brain, and then rub a wet finger down the frog's back, the creature will croak as if pleased. Frogs must be easily pleased.

